# Apoptosis-mediated inhibition of human T-cell acute lymphoblastic leukemia upon treatment with *Staphylococus Aureus* enterotoxin-superantigen

**DOI:** 10.3389/fimmu.2023.1176432

**Published:** 2023-06-12

**Authors:** Alejandra Duarte, Daniela R. Montagna, Mercedes Pastorini, Mercedes Alemán

**Affiliations:** ^1^ Institute of Experimental Medicine, National Council of Scientific and Technical Research, National Medicine Academy (IMEX-CONICET-ANM), Buenos Aires, Argentina; ^2^ Fundación Héctor Alejandro (H.A.) Barceló, Instituto Universitario de Ciencias de la Salud, Buenos Aires, Argentina

**Keywords:** enterotoxin, neoplasia, apoptosis, superantigen, lymphoma

## Abstract

Patients with relapsed T cell acute lymphoblastic leukemia (T-ALL) have limited therapeutic options and poor prognosis. The finding of efficient strategies against this refractory neoplasm is a medical priority. Superantigens (SAgs) are viral and bacterial proteins that bind to major histocompatibility complex class II molecules as unprocessed proteins and subsequently interact with a high number of T cells expressing particular T cell receptor Vβ chains. Although on mature T cells, SAgs usually trigger massive cell proliferation producing deleterious effects on the organism, in contrast, on immature T cells, they may trigger their death by apoptosis. On this basis, it was hypothesized that SAgs could also induce apoptosis in neoplastic T cells that are usually immature cells that probably conserve their particular Vβ chains. In this work, we investigated the effect of the SAg Staphylococcus *aureus* enterotoxin E (SEE) (that specifically interacts with cells that express Vβ8 chain), on human Jurkat T- leukemia line, that expresses Vβ8 in its T receptor and it is a model of the highly aggressive recurrent T-ALL. Our results demonstrated that SEE could induce apoptosis in Jurkat cells *in vitro*. The induction of apoptosis was specific, correlated to the down regulation of surface Vβ8 TCR expression and was triggered, at least in part, through the Fas/FasL extrinsic pathway. The apoptotic effect induced by SEE on Jurkat cells was therapeutically relevant. In effect, upon transplantation of Jurkat cells in the highly immunodeficient NSG mice, SEE treatment reduced dramatically tumor growth, decreased the infiltration of neoplastic cells in the bloodstream, spleen and lymph nodes and, most importantly, increased significantly the survival of mice. Taken together, these results raise the possibility that this strategy can be, in the future, a useful option for the treatment of recurrent T-ALL.

## 1 Introduction

Human T-cell acute lymphoblastic leukemia (T-ALL) is an aggressive malignant neoplasm that accounts for about 20% of all ALL and is more common in adults than in children. Survival of chemotherapy-treated patients is around 50% at 5 years. However, patients with relapsed disease have poor outcome with < 10% of patients surviving long term (1). On this basis, new and more personalized strategies against these refractory types of T-leukemia are intensely investigated but no significant therapeutic improvements have been achieved to date.

Superantigens (SAgs), the most potent bioactive molecules discovered, are bacterial and virus proteins (2–4) that bind to major histocompatibility complex (MHC) class II molecules as unprocessed proteins outside of their peptide-binding grooves and interact predominantly with the variable region domains (V) of the T-cell receptor (TCR) (5, 6). Compared to a normal antigen-induced T-cell response where 0.0001-0.001% of the body’s T-cells are activated, SAgs are capable of activating up to 20% of the body’s T-cells (7–13).

On this basis, SAgs and targeted SAgs have been used to enhance immunogenicity of murine and human tumor cells in different experimental models and clinical trials, mostly by fusing the Fab region of tumor-reactive monoclonal antibodies with superantigen staphylococcal enterotoxin A (SEA), B (SEB) or E (SEE) (14). In addition, effectiveness of SAgs might be increased by combination therapy with traditional anticancer drugs (14). However, the large numbers of SAgs-activated T-cells initiate a robust proliferation, and released large amounts of cytokines in the blood that can cause shock, and eventually death. Further, after several rounds of proliferation, SAg reactive T cells undergo apoptosis or become anergic resulting in a severely compromised immune system. As a whole, the underlying basis of the SAg-mediated antitumor strategy aimed to enhance tumor immunogenicity, is the ability to induce a massive proliferation of antitumor immune T cells.

An entirely different strategy is based on the putative capacity of SAgs to induce apoptosis rather than proliferation in neoplastic T cells through the chain variable (Vβ) region of their TCR to which each SAg binds specifically (15). This less explored proposal to date is based on the observation that responses of T-cells to SAgs depend on the maturity of the T-cell population studied. In effect, on one hand, SAgs can induce mature normal CD4 and CD8 T-cells to proliferate and produce a cytokine storm, secondarily driving mature proliferating T-cells into a state of anergy. On the other hand, SAgs can cause immature normal T-cells to become primarily depleted *via* apoptosis (16). On this basis, it was hypothesized that SAgs could also induce apoptosis in neoplastic T cells, since they are usually immature.

This strategy might be useful to treat T-leukemias and lymphomas bearing TCR with particular Vβ regions. In this context, numerous reports have described the ability of SAgs to cause apoptosis of normal immature cells expressing TCR with a specific subset of Vβ TCR (10, 12, 13, 17, 18). For instance, in normal human T cells, both in the thymus and in the periphery, SEE has an affinity for Vβ 5.1, 6.3, 6.4, 6.9, 8, while SEB has an affinity for Vβ: 1.1, 3.2, 6.4, and 15.1 (19, 20).

In previous reports, we described the capability of SAg to induce apoptosis of murine lymphoma cells *in vitro* and *in vivo*, increasing the survival of lymphoma-bearing mice (21). In the present work, in an attempt to extend our previous observations to a human model, we have investigated the apoptotic effect of SAgs on the Jurkat human neoplastic line that is considered one of the best models of the highly aggressive recurrent T-ALL, since it was established initially from the peripheral blood of a 14-year-old boy with relapsed T-ALL (ALL) (22, 23). We used Jurkat cells bearing the Vβ8 chain in its receptor and SEE, which specifically interacts with cells that express Vβ8 chain. In addition, we aimed to explore the molecular mechanisms underlying the putative apoptotic effect exerted by SAgs on Jurkat cells. We have tested the effect of SAgs on Jurkat cells implanted subcutaneously (s.c.) and intravenously (i.v.) in NSG (Nod Scid gamma) mice which are among the most immunodeficient laboratory animals described to date. The extreme immunodeficiency of NSG mice make them one of the best recipients for several types of human tumors, including both lines and primary tumor cells (24).

## 2 Materials and methods

### 2.1 Cell lines and culture

The human Jurkat T leukemia cell line, clone E6-1 (TIB-152), and human monocytic cell line THP1 (TIB-202) were obtained from the ATCC. Both cell lines were maintained at 37°C in 5% CO2 in a humidified atmosphere in RPMI 1640 culture medium supplemented with 10% FCS (GBO), 1% antibiotic-antimycotic, and 1% L-glutamine (GIBCO). THP1 cells (3 x 10^3^) were stimulated with PMA (10ng/ml) for 24 hours to differentiate into adherent cells (25) and can be used as antigen-presenting cells. Then 3 x 10^4^ Jurkat T cells were cultured with THP1 cells in the presence of SAg or PBS as controls. When indicated, Jurkat cells were treated with Caspase-9 Inhibitor III (Z-LEHD-FMK) or Caspase-8 Inhibitor II (Z-IETD-FMK) (Calbiochem) for 2 hours before SAg treatment, at a final concentration of 25µM. Also, the Fas pathway was inhibited by incubating Jurkat cells with HIgG (100µg/ml) for 1 hour and then with Fas-Fc chimera recombinant (Sigma) (10µg/ml) for 2 more hours before SAg treatment. Lymphocytes were purified from healthy human peripheral blood and were isolated by Ficoll-Hypaque gradient. Cells were seeded in plates for 2 h for adherence, and non-adherent cells (lymphocytes) were removed and maintained in culture.

### 2.2 Mice

Male NSG (NOD/SCID Gamma) (n=18) were kindly provided by Dra. Claudia Lanari from the Instituto de Biología y Medicina Experimental de Buenos Aires (IBYME). Male nude mice (12) were purchased to the Comisión Nacional de Energía Atómica (CNEA). The mice were used at 6-8 week-old and maintained under specific pathogen-free conditions based on “Guide for Care and Use of Laboratory Animals. Bethesda, MD: National Institutes of Health; 1985”. NIH publication N 85-23. Experiments were approved by the ethical committee of the IMEX-CONICET (CICUAL N° 039/2017).

### 2.3 Superantigens

Toxins S*taphylococcal enterotoxin* B (SEB) and E (SEE) were purchased from Toxin Technologies and used in a final concentration of 1µg/ml. The toxins were diluted in PBS and frozen in aliquots at -20°C until use. SEE has an affinity for Vβ: 5.1, 6, 8, and SEB affinity for Vβ: 1.1, 3.2, 6.4, and 15.1.

### 2.4 Antibodies and reagents

Cells were stained with the indicated monoclonal antibodies conjugated or not to fluorescein isothiocyanate (FITC), phycoerythrin (PE), or Cy-chrome 5 (Cy); anti-Human Fas Ligand (Fas-L), anti-human Vβ8 (BD Pharmingen) anti-human CD4 (Biocience), and/or dyes: Annexin-V (BD bioscience), Propidium Iodine (Sigma) 3,3`-diethyloxacarbocyanine iodine (DiOC_2_ (3)), 5,6 carboxyfluorescein diacetate succinimidyl ester (CFSE), (Molecular Probes, Eugene, OR, USA). Briefly, cells (1 × 10^6^) were incubated with the appropriate antibody or dye for 20 minutes at room temperature in the dark (26) and washed with PBS. For Flow cytometry analysis, 30,000 events were collected in linear mode for forward scatter and side scatter and log amplification for FL-1, FL-2, and FL-3 using a FACScan cytometer (BD Biosciences). Background values were obtained with isotype controls (BD Pharmingen). Results were analyzed by using Cell Quest software (BD Immunocytometry System).

### 2.5 Proliferation assays

Lymphocyte proliferation was evaluated by CFSE dilution. Briefly, 1 x 10^7^cells/ml was suspended in 0.3% BSA/PBS, and then CFSE was added (0.5µM) and incubated for 10 min at 37°C. Cells were washed and incubated for 5 min at 37°C between washes three times, stopping the colorant incorporation with RPMI supplemented with 15% FCS. Afterward, 1 x 10^5^ CFSE-Jurkat cells were cultured in 96-well flat-bottom plates in the presence or absence of 3 x 10^3^ THP1 cells, and SAg (1µg/ml) or PBS were added and cultured for 24, 48, or 72 h. Finally, cells were stained with anti-Vβ8-PE mAbs and evaluated by flow cytometry as described above, considering a low expression of CFSE as proliferating lymphocytes.

### 2.6 Apoptosis assays

*Annexin-V binding*. Jurkat T cells (1x10^5^) were co-cultured with THP1 cells (3 x 10^3^) in 96-well flat-bottom plates, and SAg (1µg/ml) or PBS was added and cultured for 24, 48, or 72 h. Afterward, cells were recovered, washed with PBS at 4°C, suspended in 150µl of calcium Buffer, and stained with Annexin-V (10µg/ml) (Sigma). Cells were evaluated by flow cytometry as described above, and all Annexin-V positive cells were considered apoptotic.

*DNA content*. The method of Nicoletti (27) was used with minor changes to evaluate PI incorporation. Briefly, 2.4 x 10^5^ cells were suspended in 500µl PBS and were added dropwise to 4.5 ml ice-cold 70% ethanol while vortexing. After washing, the pellet was suspended in 500 µl PBS and 5 ml DNA extraction buffer (0.2MNa2HPO4; 0.1Mcitric acid; pH 7.8). After 5 min incubation, cells were washed and suspended in 140 µl RNase A (500µg/ml) and 140µl PI (100µg/ml) and incubated for 30 min at room temperature in the dark. Samples were washed in PBS before analysis by flow cytometry. Apoptotic cells can be recognized as cells having less DNA than G1 cells (“sub-G1” peak).

### 2.7 Mitochondrial membrane depolarization

Jurkat cells were treated with SAg (10µg/ml) or PBS for 72 h. Afterward, cells were stained with DiOC_2_(3) at a final concentration of 10nM according to the manufacturer’s protocols. Samples were evaluated by flow cytometry, considering a low expression of DiOC_2_(3) cells as indicative of mitochondrial depolarization (ΔΨm). As a positive control, cells were treated in parallel samples with the carbonyl cyanide 3-chlorophenylhydrazone (CCCP) (50µM), a protonophore uncoupling agent.

### 2.8 Protein isolation and western blot

After 6 hours of treatment, Jurkat cells were pellet at 1800 rpm for 10 minutes. Then, cells were suspended in a hot 2 x SDS gel sample buffer following the manufacturer’s conditions. The extracts were separated in 15% SDS-PAGE, then electrotransferred to Immun-BlotTM PVDF Membrane (Bio-rad). The membranes were blocked with BSA 1% for 1 hour at room temperature and incubated overnight with the primary antibody at appropriate dilutions. After being washed, the membranes were incubated with horseradish peroxidase (HRP)-conjugated secondary antibodies, and immunoreactive bands were detected SuperSignal^®^ West Pico Chemiluminescent Substrate (PIERCE, Thermo Scientific). Cells were stained with anti-human BID (R&D system), anti-Human β-Actin (Cell signaling), anti-Human Bax and anti-Human Bcl-2 (BD Bioscience), mouse anti-goat, Goat-anti Rabbit, and Goat-anti-mouse (Santa Cruz Biotechnology) and subjected to western blot analysis.

### 2.9 Total RNA isolation, RT-PCR and qPCR

Total RNA was isolated using the RNA extraction KIT (Qiagen). RNA quantification was assessed with Gene Quant Pro (Amersham Biosciences). Reverse transcription was carried out using Cloned AMV Reverse Transcriptase (Invitrogen). The cDNA generated was further amplified by PCR under optimized conditions using Taq DNA Polymerase Recombinant (Invitrogen) or Sso Advanced Universal SYBR Green Supermix (BIO-RAD). Primer specifics for actin were used as housekeeping genes. To compare the number of amplified sequences produced from different RNA samples, the amplified actin product of each sample was used as an internal standard: Actin (Fw: 5´-TATGTGGGTGACGAGGCCCAGAG-3´ and Rv: 5´-TACTCCTGCTTGCTGATCCACATC-3´); Bax (Fw: 5´-GACGGGTCCGGGGAGCAGCTTG-3´ and Rv: 5´-GCCCATCTTCTTCCAGATGGTG-3´); Bcl2 (Fw: 5´-TTGAAGTGCCATTGGTAC-3´ and Rv: 5´-CCAGCCTCCGTTATCCTG-3´); Fas-L (Fw: 5’-GGATTGGGCCTGGGGATGTTTCA-3´ and Rv: 5’-TTGTGGCTCAGGGGCAGGTTGTTG-3´); Vβ8 (Fw: 5´-GCCCTCAGAACCCAGGGACT-3´); Cb (Fw: 5´-AGATCTCTGCTTCTGATGGCTC).

The Q-PCR reaction condition were one cycle of 95°C for 30 secs, followed by the 40 cycles at 95°C for 15secs and 50°C for 45 secs.

The PCR products were resolved on a 2% (wt/vol) agarose gel containing 0.5µg/ml of ethidium bromide to determine the molecular sizes of different amplicons. The gel images were acquired with the GelPro analyzer (IPS, North Reading, MA). The levels of different amplicons mRNA were quantitated using a computer-assisted image analyzer (ImageQuant 5.2). The PCR results for each sample were normalized by actin mRNA as an internal control.

### 2.10 Xenograft tumor model

Ten NSG mice were inoculated subcutaneously with 1 x 10^6^ Jurkat cells. After three weeks, the mice were treated intraperitoneally with SEE (10μg) or PBS, and the tumor size was measured once a week for 6 weeks. According to the Attlia and Weiss formula (28), the tumor volume was calculated using a caliber to make the measurements. The formula calculated tumor volume in mm3: volume = a x b^2^ x 0.4, where a and b are the major and minor diameters, respectively. This formula allows calculating the equivalent of the tumor mass without the euthanasia of the animals. The blood was collected (100μl) by the submandibular venipuncture collection method once a week for 6 weeks. A calibrated coulter measured the analysis of the cellular component of the blood.

### 2.11 Survival experiments

To investigate whether bacterial SAg improved the survival of mice carrying Jurkat cells, 10 x 10^6^ cells were inoculated into the tail vein of NSG (n=18) or nude (n=12) mice. On day 2, the mice were intraperitoneally treated with 50µg of SEE or PBS. All survival studies were conducted in a blind and random fashion. Animals were monitored daily for general appearance and weight change. Mice showing signs of pain and suffering were considered having achieved their end points and were euthanized. Day of death was considered the day at which each mouse was euthanized. Infiltration of Jurkat cells (metastases) to lymph nodes, spleen, and bone marrow was examined by evaluating the presence of Vβ8+ lymphocytes by cytometry.

### 2.12 Statistical analysis

Statistical significance levels were determined using Student’s t-test or variance analysis (ANOVA) followed by the Tukey tests. Values were expressed as mean ± SE. Differences were considered significant whenever the *p-*value was ≤ 0.05 and a power analysis of 95%. A comparison of survival curves was performed using the Log-rank test with Prism software. Survival rates were analyzed by the χ^2^ test.

## 3 Results

### 3.1 SEE induces apoptosis and reduces Vβ8 expression in Jurkat cells *in vitro*

**Figure 1:**
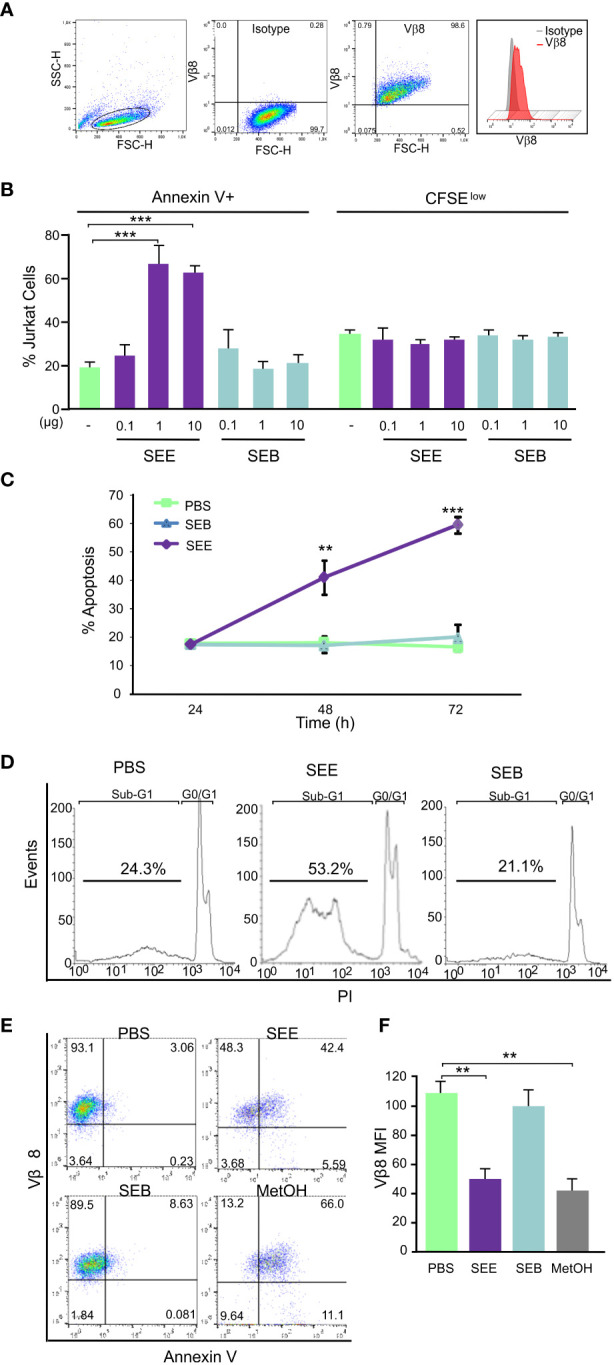
SEE induces dose-dependent apoptosis in Jurkat cells without promoting proliferation. Jurkat cells were treated with SEE (1µg/ml), SEB (1µg/ml), or PBS in the presence of THP1 cells for 72 h. **(A)** Population of Jurkat cells and the percentage of Vβ8+ cells **(B)** *Left:* percentage of cells expressing Annexin-V (***p<0.001 SEE vs. PBS); *Right:* percentage of cells expressing low content of CFSE **(C)** Percentage of Annexin-V+ cells (***p<0.001 SEE vs. PBS) **(D)** Representative histogram showing apoptotic cells having less DNA content than G1 cells (“sub-G1” peak) **(E)** Representative dot plot showing the percentage of Vβ8+/Annexin-V+ cells incubated with PBS, SEE, SEB or 3% MetOH **(F)** Mean fluorescence intensity of Vβ8 Expression (**p<0.01 SEE vs. PBS; **p<0.01 MetOH vs. PBS). In all experiments 30,000 events were collected by FACScan flow cytometer and data are presented as mean ± SEM of three independent experiments.

It has been described that SAg can induce apoptosis in murine lymphoma T cells *in vitro* and *in vivo* (21). In order to evaluate whether SAg could induce apoptosis in human neoplastic cells, Jurkat cells which express Vβ8 (**Figure 1A**) were cultured together with human monocytic cell line THP1 as presenting cells and then cells were stimulated with a Vβ8-specific (SEE) or with non-specific SAg (SEB). Results show that SEE but not SEB induced apoptosis of Jurkat cells measured by Annexin-V+ in a dose- (**Figure 1B**) and time-dependent manner (**Figure 1C**), whereas no differences in cell proliferation were observed at the same conditions (**Figure 1B**). We also evaluated primary CD4+ T cells from healthy individuals and found that SAgs did not induce apoptosis or proliferation in these cells (**Supplementary Figure 1**). Besides, the DNA content was measured by PI incorporation, wherein cells having less DNA than G1 cells (“sub-G1” peak) were identified as apoptotic cells (**Figure 1D**). It’s worth noting that the treatment with SEE led to a reduction in Vβ8 expression in Jurkat cells. This observation suggests that the phenomenon could be associated with cell death, as a similar decrease in Vβ8 expression was seen when apoptosis was induced with 3% methanol (**Figures 1E, F**). Together, these results suggest that SAg-induced apoptosis of human T cells bearing the Vβ region is specific and does not involve lymphocyte proliferation.

### 3.2 Mechanisms involved in SEE-induced apoptosis in Jurkat cells *in vitro*

**Figure 2:**
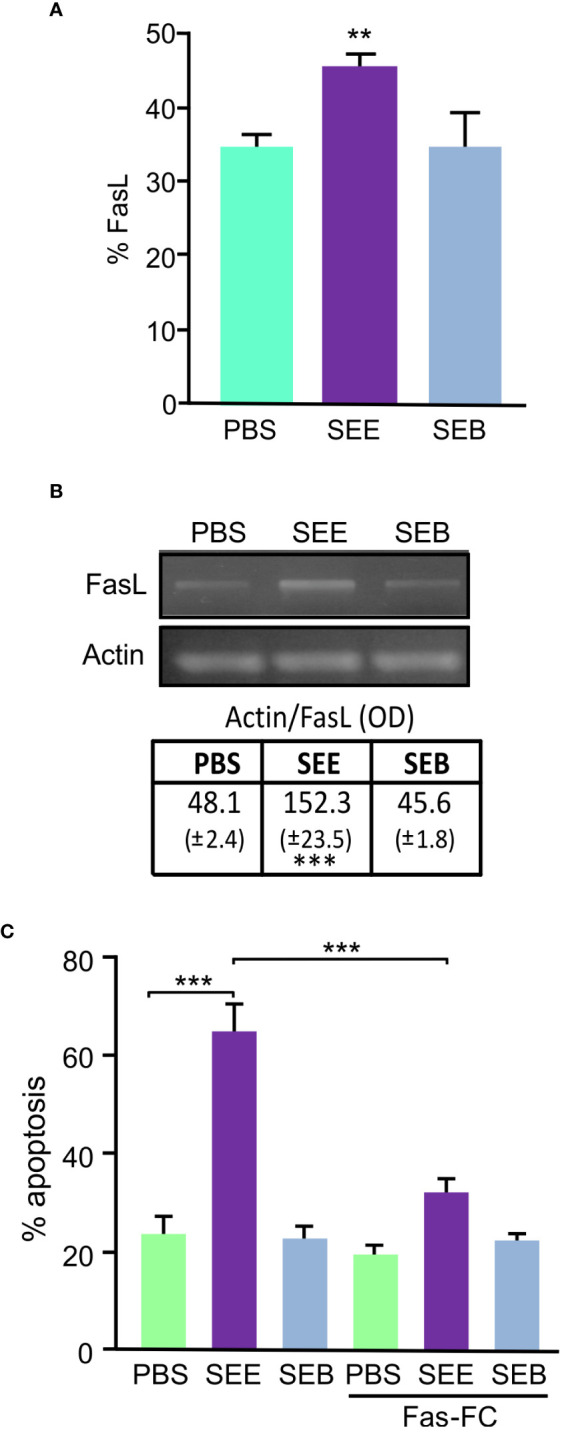
SEE induces apoptosis through the Fas-FasL pathway. Jurkat cells were treated with SEE (1µg/ml), SEB (1µg/ml), or PBS in the presence of THP1 cells for 72 h. **(A)** Percentage of FasL expression; 30,000 events were collected by FACScan flow cytometer (**p<0.01 SEE vs. PBS). **(B)** Representative picture of an RT-PCR showing FasL transcript and its quantification after 18 hours of culture (***p<0.001 SEE vs. PBS). **(C)** The percentage of apoptotic Jurkat cells was measured with or without Fas-Fc treatment prior to SAg exposure (***p<0.001 SEE vs. PBS; ***p<0.001 SEE vs. SEE+Fas-Fc). Data are presented as the mean ± SEM of three independent experiments.

Apoptosis can be triggered by extrinsic signals transduced through cell surface receptors; in particular, Fas Ligand (Fas-L) is activated in T cells after stimulation through the TCR (29). Previous studies have demonstrated the involvement of Fas/Fas-L interaction in bacterial and viral Sag mediated apoptosis of murine T lymphomas. We aimed to investigate whether SEE SAg induces apoptosis of Jurkat cells through the Fas/Fas-L pathway. We analyzed the membrane expression and mRNA of these molecules in Jurkat cells treated with SEE, SEB, or PBS and found a significant increase in Fas-L protein (**Figure 2A**) and Fas-L mRNA (**Figure 2B**) expression in Jurkat cells. However, Fas values did not vary with any treatment (data not shown). Furthermore, when cells were incubated with a Fas-Fc construction, a competitive inhibitor of Fas/Fas-L interactions, SAg-induced apoptosis was reduced by about 47% (**Figure 2C**), suggesting that SEE SAg triggers lymphocyte apoptosis through the Fas/FasL pathway by inducing Fas-L expression in lymphocytes.

**Figure 3:**
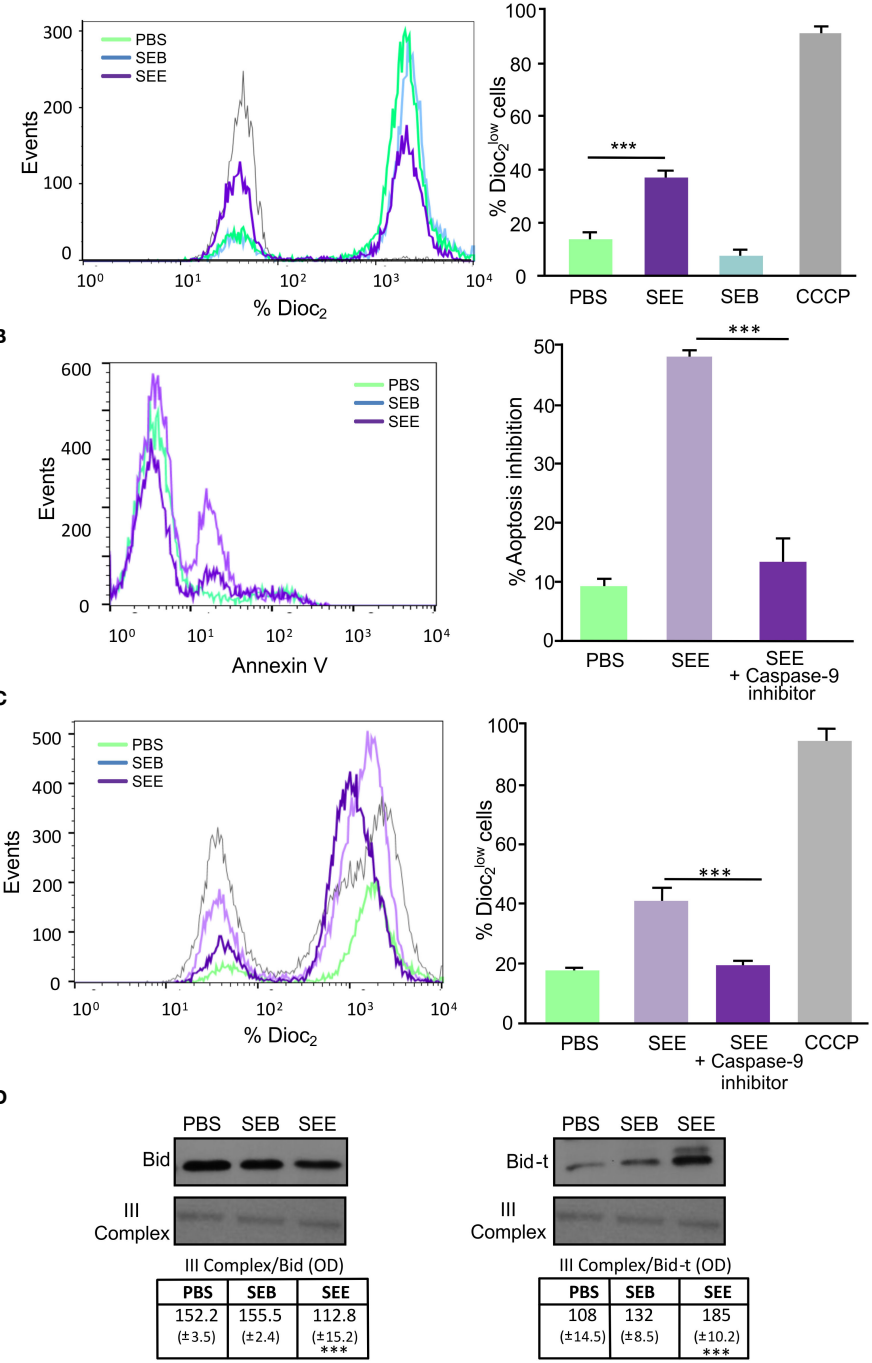
SEE activates the mitochondrial pathway and induces apoptosis by cross-talk between the intrinsic and extrinsic pathways. Jurkat cells were cultured for 72 hs in the presence of THP1 cells with PBS, SEE (1µg/ml), or SEB (1µg/ml). **(A)** Representative histogram and quantification showing changes in the mitochondrial membrane depolarization measured as ${\text{Dioc}}_{2}^{\text{low}}$ expression (***p<0.001 SEE vs. PBS); CCCP was used as a positive control. **(B)** Representative histogram of Annexin-V expression and percentage of apoptosis inhibition in Jurkat cells incubated with Z-IETD-FMK (caspase-9 inhibitor) or DMSO before the treatment with SEE or PBS (***p<0.001 SEE + caspase-9 inhibitor vs. SEE). **(C)** Representative histogram and bar graph showing changes in the mitochondrial membrane depolarization (% ${\text{Dioc}}_{2}^{\text{low}}$ expression) in Jurkat cells incubated with Z-HETD-FMK (caspase-8 inhibitor) or DMSO before treatment with SEB, SEE, or PBS. (***p<0.001 SEE + caspase-8 inhibitor vs. SEE). **(D)** Representative Western blot images are presented and the optical density of Bid and Bid-t bands were quantified and normalized to III-Complex. The relative levels are displayed in the graph (***p<0.001 SEE vs. PBS). Data are presented as the mean ± SEM of three independent experiments.

Changes in the mitochondrial membrane potential were evaluated to investigate whether SEE activates the intrinsic death pathway. The percentage of cells which expressed low DiOC_2_(3) was indicative of mitochondrial depolarization degree. We observed a DiOC_2_(3)^low^ increase in SEE-treated Jurkat cells, but no differences were observed in the cells treated with SEB (**Figure 3A**).

Moreover, when Jurkat cells were incubated with a Caspase-9 inhibitor, a significant decrease in apoptosis was observed (**Figure 3B**), suggesting that SEE triggers lymphocyte apoptosis through the intrinsic pathway. However, Fas can also induce mitochondrial events amplifying the signal through death receptors of the intrinsic pathway (30). This is because Caspase 8 could cleave Bid, a protein whose truncated form (Bid-t) can induce mitochondrial membrane depolarization (31). Notably, when Jurkat cells were incubated with a Caspase-8 inhibitor before SAg treatment, a decrease in DIOC_2_(3)^low^ cells were observed (**Figure 3C**). To assess whether Bid was concerned with SEE-induced apoptosis, whole cell extracts were compared by western blot analysis, and results showed that SEE caused a decrease in full–length Bid along with the detection of the Bid-t in Jurkat T cells (**Figure 3D**), thus demonstrating that SEE induces apoptosis through both cell death pathways interrelated. However, we do not rule out the direct involvement of the intrinsic pathway.

### 3.3 Anti-neoplastic effect of SAg *in vivo*

#### 3.3.1 The SEE suppresses tumor growth in NSG mice

**Figure 4:**
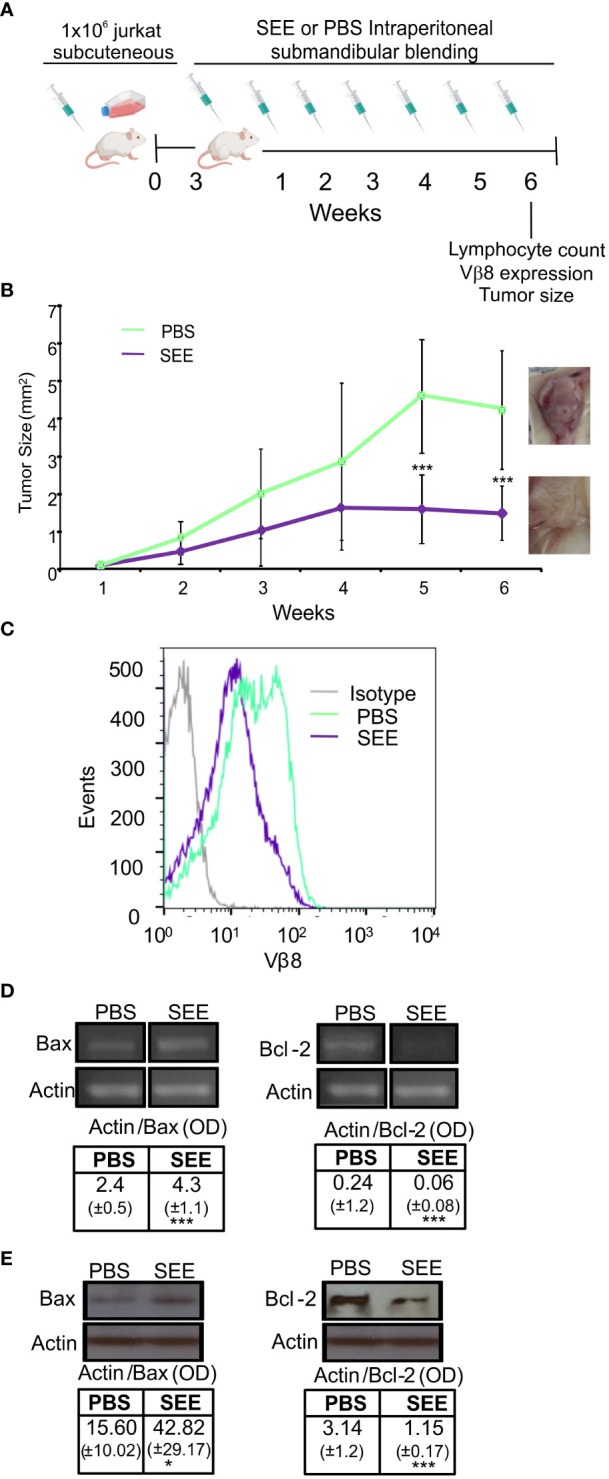
The SEE reduces tumor size in mice by inducing apoptotic. **(A)** Experimental scheme **(B)** Tumor volumes (mm3) were measured weekly in the control and SEE treatment groups for six weeks (**p<0.01; PBS vs. SEE; n=6). **(C)** Representative histogram showing the expression of the specific Vβ8 chain of TCR in tumor cells from control and SEE treated groups at the final time point after six weeks **(D)** A representative image and quantification of the expression levels of Bax and Bcl-2 measured in tumor cells from control and SEE treated groups at the final time point after six weeks using RT-PCR. Optical density (OD) values are presented **(E)** Representative images of Western blots for Bax and Bcl-2 expression levels are presented. The optical density of the bands was quantified and normalized to Actin. The relative levels are shown. (Bax: *p<0.05 SEE vs. PBS; Bcl-2: ***p<0.001 SEE vs. PBS). Data are presented as the mean ± SEM of three independent experiments.

To determine the anti-neoplastic effect of SEE *in vivo*, we employed a xenograft model of Jurkat cells in NSG mice to quickly screen tumor bulk and lymphocyte infiltration. Twelve NSG mice were subcutaneously (s.c.) inoculated with (1 x 10^6^) Jurkat cells, and 3 weeks later, when solid tumor mass was detectable, six mice were treated intraperitoneally (i.p.) with SEE and the other 6 with PBS (**Figure 4A**). After that, tumor growth was measured once a week for 6 weeks. We found that tumor growth inhibition started to be significant from the fifth week in those mice treated with SEE (**Figure 4B**). As expected, Vβ8 expressions in tumor cells were significantly reduced in SEE-treated mice (**Figure 4C**). In addition, we evaluated whether those apoptotic features exerted by SEE *in vivo* reflected that observed *in vitro* by assessing the members of the Bcl-2 family. **Figure 4D** shows that SEE treatment resulted in a substantial increase in Bax mRNA levels and a decrease in Bcl-2 mRNA levels in tumor cells. This effect was also observed in the levels of the respective proteins (**Figure 4E**).

#### 3.3.2 SEE reduces the infiltration of neoplastic cells in the bloodstream

**Figure 5:**
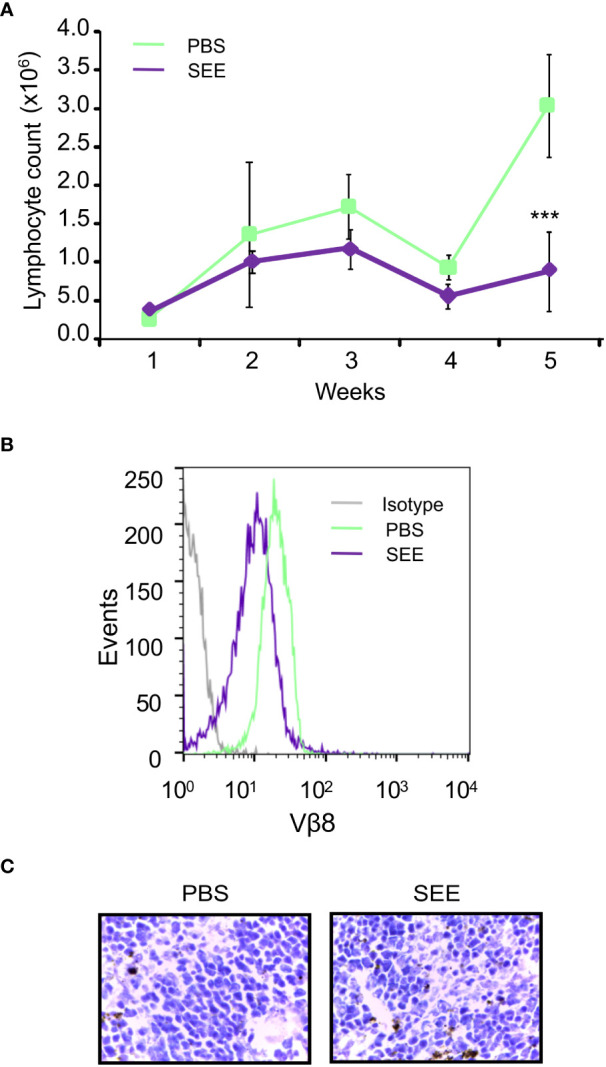
SEE reduces the number of lymphocytes in the blood of mice with intradermally xenografted Jurkat cells. **(A)** Lymphocyte counts were measured in blood samples once a week for six weeks using a Coulter counter (**p<0.01; PBS vs. SEE; n=6). **(B)** Representative histogram showing the expression of the specific Vβ8 chain of TCR in the blood of the control and SEE treatment groups was generated at the final time point after six weeks **(C)** H&E stain of mouse spleen demonstrated no significant pathological changes between the control and SEE treatment group.

In order to assess whether SEE treatment could prevent the infiltration of lymphocytes into the bloodstream, mice were submandibulary bled once a week for 6 weeks after SEE or PBS injection. Then, blood lymphocytes were evaluated by flow cytometry and hematologic coulter. As can be seen in **Figure 5A**, SEE treatment elicited a decrease in neoplastic lymphocyte infiltration in blood, which correlated with a down-regulation of Vβ8 TCR expression (**Figure 5B**). In contrast, the rest of the blood cell populations were not altered (**Figure 5C**; **Table 1**). These results demonstrate that apoptosis of neoplastic cells induced by SAg in the tumor reduces neoplastic cell infiltration in the periphery.

**Table 1:** Quantification of the respective blood population measured by coulter in control and SEE treatment groups at the final time after 6 weeks without significant differences (ns *p*>0.05; PBS vs. SEE; n=6).

		RBC (x10^6^)	Monocytes (x10^6^)	Neutrophils (x10^6^)	PLT (x10^6^)
Week 1	PBS	8.21 ± 4.64	2.50 ± 7.07	1.83 ± 4.95	9.31 ± 2.35
	SEE	8.20 ± 6.51	2.03 ± 5.77	1.47 ± 1.82	8.87 ± 2.35
Week 2	PBS	7.19 ± 4.27	2.50 ± 2.12	3.80 ± 1.49	2.53 ± 3.76
	SEE	7.19 ± 5.12	2.50 ± 7.07	3.00 ± 5.69	2.46 ± 3.76

RBC, red blood cells; PLT, platelet.

#### 3.3.3 SEE increases the survival of mice bearing neoplastic cells

**Figure 6:**
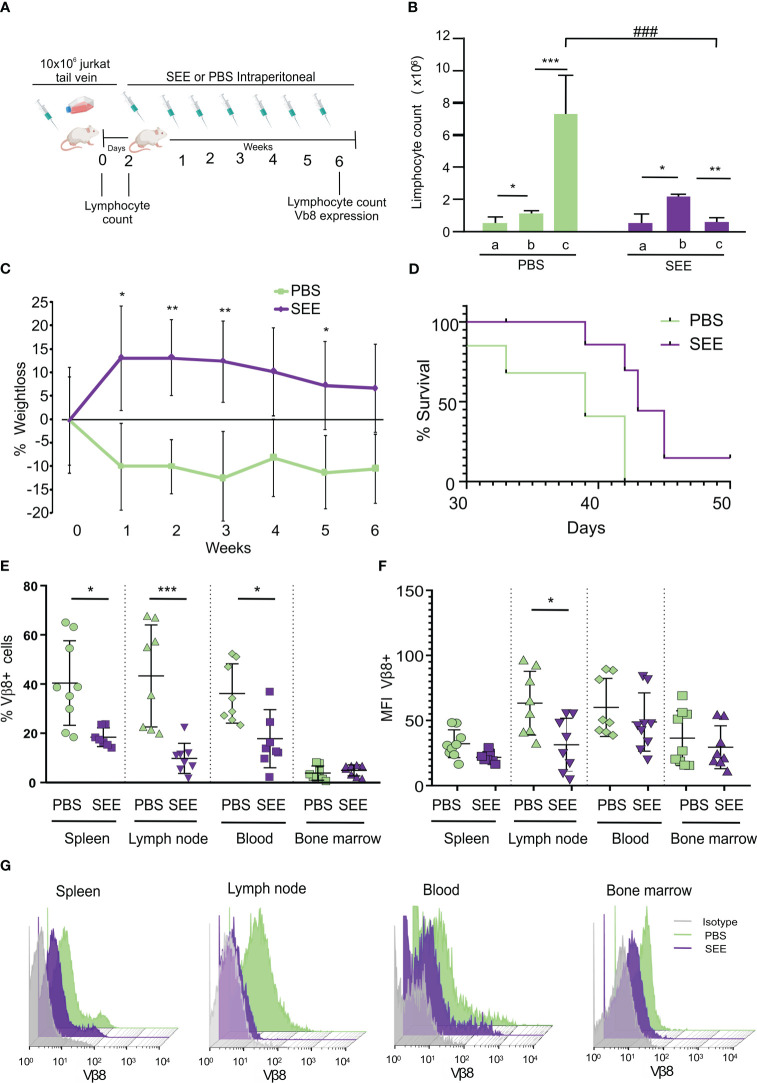
SEE increases mice survival. **(A)** Experimental scheme **(B)** Lymphocyte counts in mouse blood were measured before inoculation of Jurkat cells (a), two days after inoculation (b), and at the final time point after treatment (c). The results are presented as a quantification of lymphocytes (*p<0.05; PBS a vs. PBS b; ***p<0.001; PBS b vs. PBS c; *p<0.05; SEE a vs. SEE b; **p<0.01; SEE b vs. SEE c; ^###^ *p*<0.001; PBS c vs. SEE c; n=9). **(C)** Weight loss curve for 6 weeks (*p<0.05; PBS vs. SEE; **p<0.01; PBS vs. SEE). **(D)** Survival curve of mice treated with PBS or SEE after inoculation of Jurkat cells (***p<0.0001; PBS vs. SEE; n=9) **(E)** Percentage of Vβ8+ cells in spleen, lymph node, blood, and bone marrow from mice treated either with PBS or SEE (spleen: *p<0.05 PBS vs. SEE; lymph node: ***p<0.001 PBS vs. SEE; Blood: *p<0.05 PBS vs. SEE). **(F)** mean fluorescence intensity of Vβ8 expression in cells from spleen, lymph node, blood, and bone marrow from mice treated with PBS or SEE; (*p<0.05 PBS vs. SEE, lymph node). **(G)** Representative histogram of Vβ8 expression in mice treated with PBS or SEE in the spleen, lymph node, blood, and bone marrow cells. Data are presented as the mean ± SEM, n=9.

Thereafter, we evaluated whether SAg could improve the survival of mice carrying human neoplastic T cells simulating the clinical presentation of the disease. To this aim, 10x10^6^ Jurkat cells were intravenously inoculated (i.v.), and 2 days later, mice started to be treated with SAg (n=9) or PBS (n=9) every 7 days (**Figure 6A**). The presence of lymphocytes in blood was checked throughout the experiment. The results demonstrate that SEE-treated mice did not experience an increase in lymphocyte numbers; in fact, there was a significant decrease compared to the non-treated mice (**Figure 6B**; **Supplementary Figure 2**).

In addition, non-treated mice showed a higher weight loss (**Figure 6C**) which anticipated their accelerated deterioration and death (namely, the day at which mice achieved their end point and were euthanized) (**Figure 6D**). In effect, at day 42, while all control mice had died, about 70% of treated mice still remained alive. At the endpoint, the number of Vβ8+ cells was significantly higher in untreated mice compared with SAg SEE-treated mice in spleen, lymph nodes, and peripheral blood while no significant differences were observed in bone marrow (**Figure 6E**) suggesting that, upon the schedule of SAg treatment used herein, neither leukemic nor normal immune cells were induced to proliferate. Besides, in those SEE-treated mice with residual neoplastic lymphocytes, a down-regulation of Vβ8 TCR expression could be observed, suggesting that apoptosis had already been triggered (**Figures 6F, G**). To investigate the possible mechanism of immunological evasion related to the loss of Vβ8 in Jurkat cells after SEE treatment, we measured the expression level of Vβ8 mRNA by qPCR in a pool of Jurkat cells isolated from the spleen, blood, and lymph nodes of mice at the end of the experiment (42 days after intravenous injection). The results demonstrate that the expression of Vβ8 mRNA was significantly lower in treated mice than in non-treated mice. These findings suggest that SEE treatment reduces tumor cells *in vivo*, which is consistent with the higher survival rate observed (**Supplementary Figure 3**). No signs of toxicity associated with the treatment with SAg SEE were observed. These results indicate that SAgs can significantly extend the survival of mice by inducing apoptosis of the human neoplastic T lymphocytes restricted to a specific Vβ chain without producing overt undesired side-effects.

We also employed nude mice to evaluate the effect of the SAgs. Nude mice exhibit strong B cell and NK cell responses, which prevent tumor development in the xenograft model (data not shown). Nevertheless, to evaluate the impact of myeloid suppressor cells, which can promote tumor cell survival during chemotherapy and radiotherapy through various mechanisms, survival assays were conducted. Our findings indicate that untreated nude mice rapidly lost weight (**Supplementary Figure 4A**), with a 40% survival rate (**Supplementary Figure 4B**). Notably, these mice also exhibited an increased number of Vβ8+ cells in the spleen, lymph nodes, and peripheral blood (**Supplementary Figures 4C, D**). These results highlight the physiological significance of the antineoplastic effect of SEE, which remains significant even in the presence of other resident myeloid cells.

## 4 Discussion

Patients with relapsed T-ALL have limited therapeutic options and an extremely poor prognosis. Although a variety of salvage chemotherapy regimens have been proposed, response rates are highly unsatisfactory and in consequence, this situation represents an urgent and pivotal clinical need (1, 32, 33).

An immunological approach, based on the ability of superantigens to active and to induce the proliferation of a large number of mature T lymphocytes, might fill that medical need as far as SAgs could enhance the immunogenicity of many murine and human tumors - eventually including relapsed T-ALL - mainly by fusing them with the Fab region of tumor-reactive antibodies. In fact, in the last few years, SAgs, SAg-like proteins and SAg derivatives have been extensively investigated as immune-modifiers in the treatment of cancer, either alone or in combination with classical antitumor drugs. However, the potentially powerful antitumor activity of SAgs is limited by their intrinsic high toxicity resulting from the production of high levels of circulating cytokines by the large number of SAgs-activated and proliferating T lymphocytes (14, 34–36).

In order to circumvent these limitations, in this paper, we have taken advantage of another property of SAgs, namely their ability to induce apoptosis in certain subsets of immature T cells through specific Vβ regions which determined SAg interacts with. Assuming that neoplastic cells are usually immature ones, this strategy was aimed to inhibit not any cancer cell but neoplastic T cells bearing specific Vβ regions just as primary and relapsed T-ALL cells are supposed to bear. Some years ago, this approach proved to be effective to inhibit spontaneous murine lymphomas of T-origin (21). However, up to date, it has not been much explored despite the fact that apoptosis plays a critical role in developing T lymphocytes, both in the generation of their functional competence in the thymus and the regulation of T cell populations in the periphery (5, 37–39).

On this basis, we investigated the effect of SAgs on human neoplastic T cells in both *in vitro* and *in vivo* settings. As target, we used the Jurkat cell line derived from a recurrent T-ALL bearing the Vβ8 region in its T receptor, and as effector, we utilized the SEE SAg which specifically interacts with cells that express the Vβ8 region. Our results demonstrated that SEE could induce apoptosis in Jurkat cells grown in culture and also implanted subcutaneously and intravenously in NSG mice. NSG are one of the most immunodeficient laboratory animals described to date. This condition not only makes NSG mice to be good recipients for many transplantable human cancers but also allows faithful reproduction of their clinical course, meaning that the observations carried out in these mice might be of predictive value for clinical cases.

The induction of apoptosis in Jurkat cells was specific since it occurred when we used Vβ8-specific SAg, SEE, but not a non-specific one (SEB) that does not interact with Vβ8 region. The onset of the intracellular apoptotic signalling was correlated to the down regulation of surface Vβ8 TCR expression and did not require cell proliferation, suggesting that apoptosis mediated by SEE was not a proliferation-dependent event but a primary effect of SEE upon interaction with Vβ8-region bearing leukemic cells. In our model, apoptosis was triggered through the Fas/FasL extrinsic pathway by inducing Fas-L expression in Jurkat cells. Fas/FasL interaction is mainly responsible for eliminating chronically stimulated lymphocytes and maintaining peripheral immunological tolerance and it has been implicated in drug- and stress-induced apoptotic death of lymphocytes (39–45). However, the fact that inhibition of Caspase-9 reduced significantly both mitochondrial membrane depolarization and SEE-induced apoptosis of Jurkat cells, suggest that the intrinsic pathway of apoptosis may also be involved (46–48).

The apoptotic effect induced by SEE in Jurkat cells was therapeutically relevant. In effect, SAg treatment reduced dramatically tumor burden when leukemic cells were subcutaneously implanted in NSG mice, it decreased the infiltration of neoplastic cells in the bloodstream, spleen and lymph nodes and, most importantly, it increased significantly the survival of mice that had received leukemic cells by the intravenous route in an attempt to mimic the natural course of the disease. All of these effects were achieved without the number of either leukemic or normal lymphocytes having increased neither in blood, nor in spleen nor in lymph nodes. Actually, that number decreased in SEE-treated mice as compared with non-treated ones, meaning that the schedule of SAg used in this work prevented proliferation of leukemic and would not promote the proliferation of normal mature T cells that is a deleterious side-effect of many of the conventional treatment protocols with SAgs. Taken together, these results raise the possibility that this strategy can be, in the future, a useful and secure option for the treatment of recurrent T-ALL.

## Data availability statement

The original contributions presented in the study are included in the article/**Supplementary Material**. Further inquiries can be directed to the corresponding author.

## Ethics statement

The animal study was reviewed and approved by Institute of Experimental Medicine – National Council of Scientific and Technical Research (IMEX-CONICET). CICUAL 039/2017. All procedures involoving human samples were conducted in adherence to the ethical guidelines outlined in the World Medical Association Declaration of Helsinki. The research protocol was approved by the Ethics Committee of the National Academy of Medicine in Buenos Aires, and written informed consent was obtained from all healthy volunteers participating in the study.

## Author contributions

All authors contribute to the study conception and design. AD, DM and MP performed the methodology and proper research. Formal analysis, validation of the results of experiments was performed by AD and DM. AD, was Funding acquisition and project administration. AD and MA Wrote the manuscript edited the manuscript and prepared figures. MA Supervised the investigation. All authors contributed to the article and approved the submitted version.
