# Supplementary figures and images for: Apoptosis-mediated inhibition of human T-cell acute lymphoblastic leukemia upon treatment with Staphylococus Aureus enterotoxin-superantigen
Source: Front Immunol. 2023 Jun 12;14:1176432. doi: 10.3389/fimmu.2023.1176432 (PMC10291079; doi:10.3389/fimmu.2023.1176432)

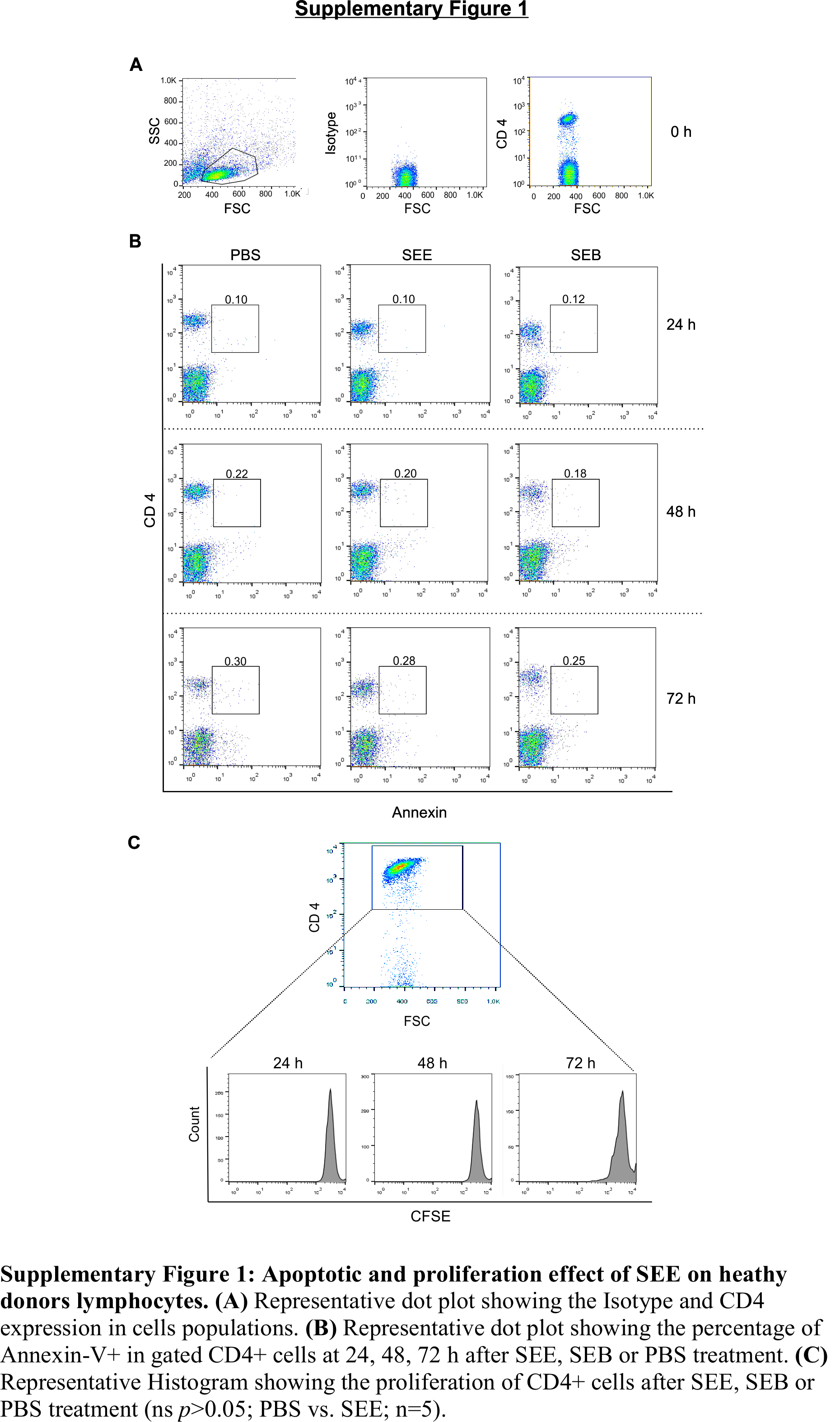

Supplement: Supplementary file 1 [file Image_1.tif]

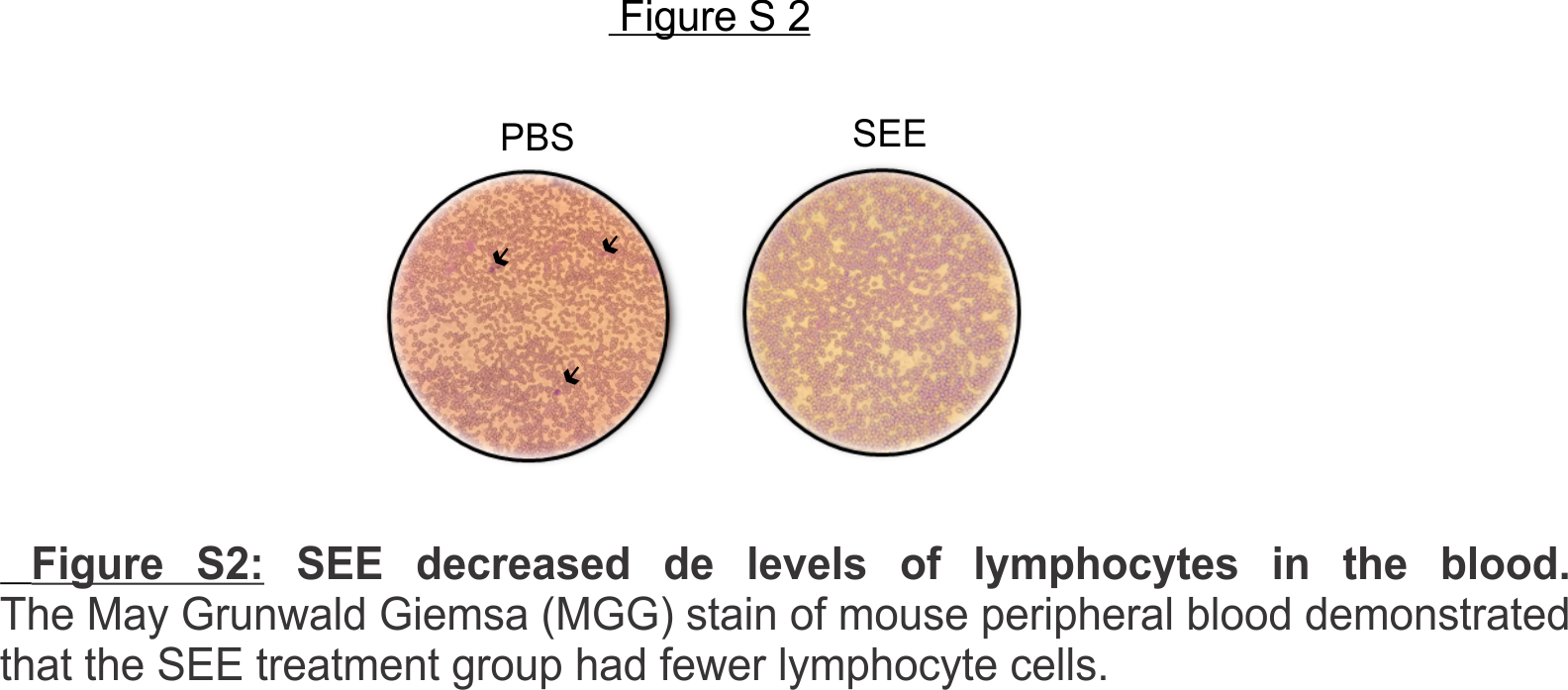

Supplement: Supplementary file 2 [file Image_2.tif]

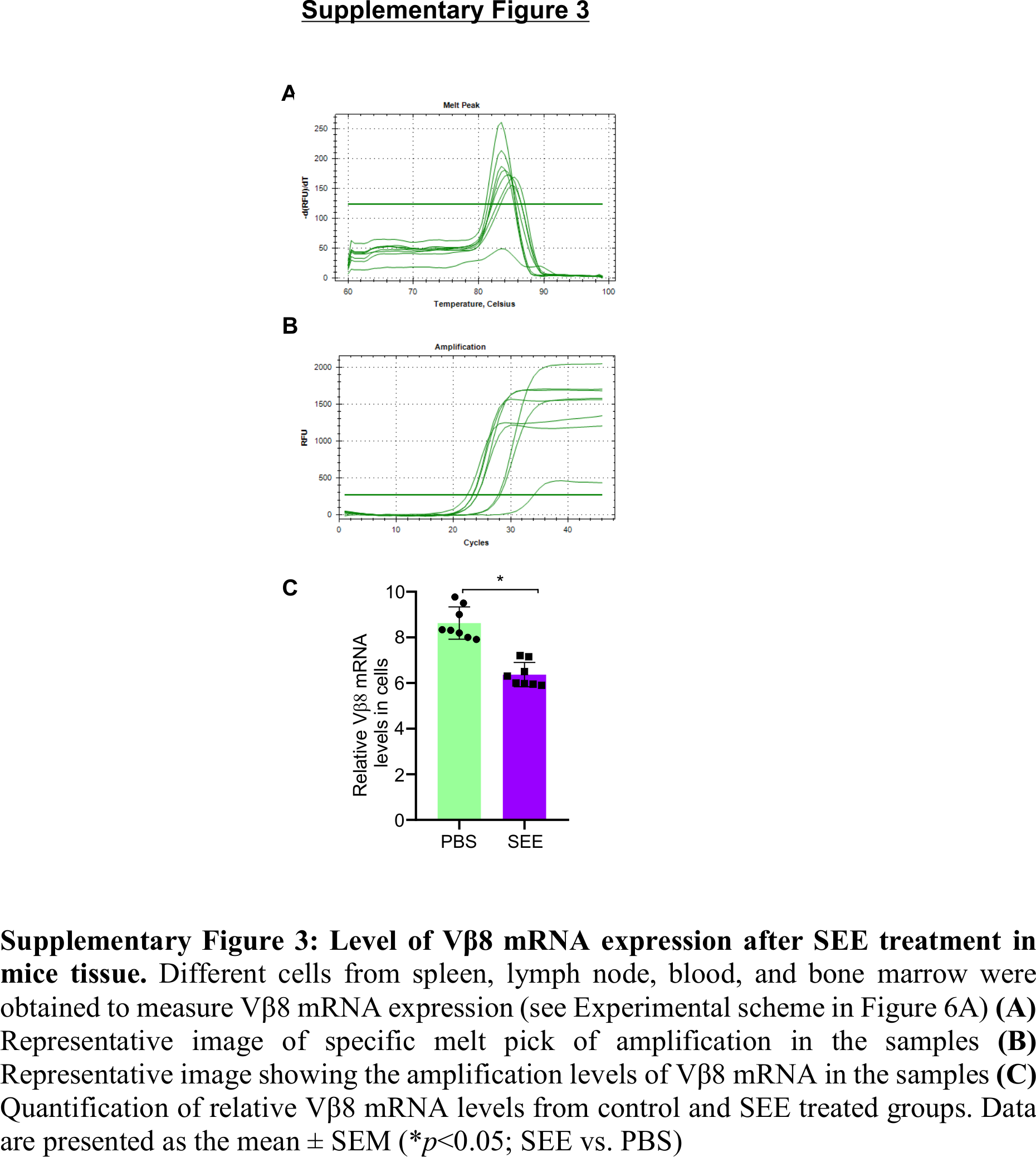

Supplement: Supplementary file 3 [file Image_3.tif]

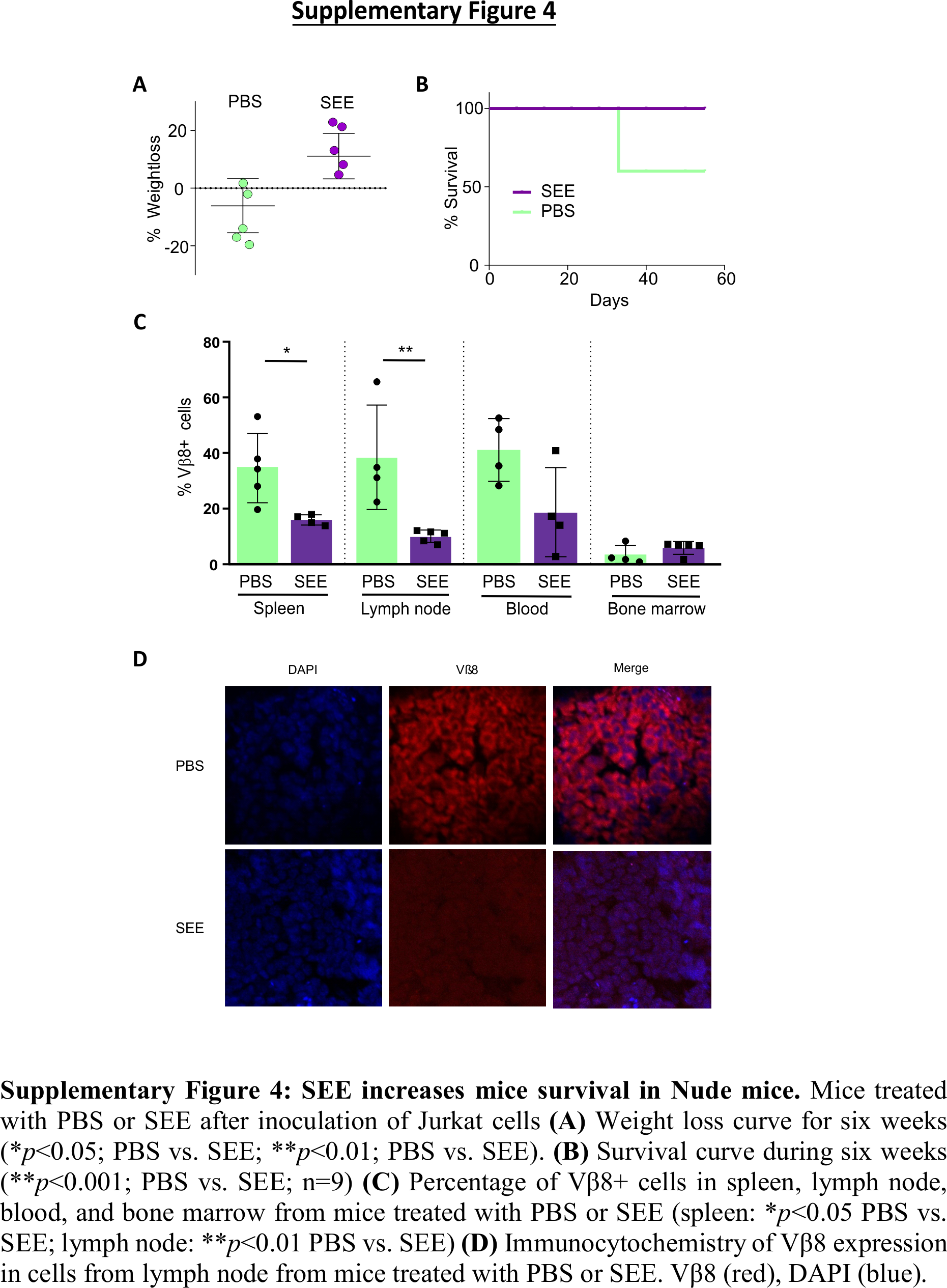

Supplement: Supplementary file 4 [file Image_4.tif]
